# Data for direct chemical deposition of PbS on chemical vapor deposition grown-graphene for high performance photovoltaic infrared photo-detectors

**DOI:** 10.1016/j.dib.2020.106273

**Published:** 2020-09-04

**Authors:** Emmanuel K Ampadu, Jungdong Kim, Eunsoon Oh, Dong Yun Lee, Keun Soo Kim

**Affiliations:** a Department of Physics*,* Chungnam National University*,* Daejeon*,* Republic of Korea; b Department of Physics and Graphene Research Institute-Texas Photonics Center International Research Center (GRI-TPC IRC)*,* Sejong University*,* Seoul*,* Republic of Korea

**Keywords:** Lead sulfide, Fourier transformed infrared spectroscopy, Heterostructure, Schottky junction

## Abstract

Over the past decades, graphene has attracted much attention from the scientific community due to its broad applications in the optoelectronics industries [1]. Owing to graphene's high transmission and high electrical conductivity, diverse functional materials/graphene hybridized heterostructures and interfaces are under extensive investigation to satisfy the increasing interest in the need for bendable, flexible and high performance optoelectronic devices [2]. Due to the good atomic lattice structure of graphene, varying heterostructures have been formed by depositing different functional materials directly on graphene [3], [4], [5].

We fabricated a vertical photovoltaic type G/PbS/Ti device by making use of the Ti/PbS Schottky junction and discussed the photocurrent transient characteristics. Lead sulfide (PbS) was deposited directly on large area CVD (Chemical vapor deposition) graphene by CBD (Chemical bath deposition). Temperature dependent photocurrent spectra of our G/PbS/Ti photovoltaic devices were measured by a Fourier transformed infrared (FTIR) set-up.

In this paper, we present the experimental procedures and the raw experimental data for the direct chemical deposition of PbS on CVD-graphene for high performance photovoltaic infrared photo-detectors. The manuscript is already available [6].

**Specifications Table**

**Table:** 

Subject	Surfaces and Interfaces
Specific subject area	PbS photovoltaic infrared photo-detectors
Type of data	Image Figure Graph
How data were acquired	XRD, SEM, FTIR, Picoammeter (Keithley 2400).
Data format	Analyzed Raw
Parameters for data collection	PbS was deposited at room temperature conditions. The samples were Pt coated prior to SEM images being taken. XRD were taken under room temperature conditions. Photocurrent was measured with a picoammeter (Keithley 2400) at room temperature. Temperature dependent photocurrent spectra were recorded with a FTIR set-up.
Description of data collection	PbS films were characterized structurally using x-ray diffractometer (XRD, SmartLab 9 KW, Rigaku) having Cu Kα radiation. The surface morphologies were observed by using cold type field emission scanning electron microscope (FESEM, S-4800, Hitachi High-Technologies). Time dependent photocurrent was measured with a picoammeter (Keithley 2400) connected to a computer with LabView^TM^ (version 8.6) Photocurrent spectra were measured at 0 V using a Bruker Vertex 80 V Fourier transform infrared (FTIR) spectrometer equipped with a globar mid-infrared light source along with a KBr beam splitter. The modulation frequency used for the photocurrent measurement was 20 kHz. For temperature dependent FTIR measurements, samples were mounted inside a vibration-free closed-cycle He optical cryostat whose bottom part with optical windows is vacuum tightly sealed in the FTIR equipment.
Data source location	Institution: Chungnam National University City/Town/Region: Daejeon Country: South Korea
Data accessibility	Hosted with this article as supplementary data
Related research article	E.K. Ampadu, J. Kim, E. Oh, D.Y. Lee, K.S. Kim, Direct chemical deposition of PbS on CVD-graphene for high performance photovoltaic infrared photo-detectors, *Mater. Lett.,* 277(2020), 128323

## Value of the Data

• Graphene tends to peel off from glass substrates when immersed in solutions containing chemicals such as NaOH. These data are important as we describe direct deposition of PbS on graphene from a solution containing 570 mM of NaOH. The deposition was successful after the annealing of graphene/glass.
• Scientific researchers who want to chemically synthesize materials directly on graphene can benefit from our data.
• These data can be helpful for further studies using graphene and PbS for flexible infrared devices taking advantage of the high transmission of graphene in the infrared spectral range.
• We provide detailed experimental processes for direct chemical synthesis of PbS on graphene.
• Photocurrent transient behaviours have been attributed to the accumulation of photo-generated carriers at interfaces and back injection of the carriers. These explanations can be extended to the photocurrent transient behaviours of other heterostructures.

## 1 Data Description

This work presents a step-by-step procedure and the raw experimental data for direct deposition of PbS on large area CVD-graphene for high performance photovoltaic infrared photo-detectors. This article provides all the raw data in reference [6] in excel spreadsheets; datasheet 1 is the XRD of PbS deposited on graphene and the transmission spectra of graphene, datasheet 2 is the time trace of photocurrent from our graphene/PbS/Ti device and datasheet 3 is the recorded FTIR spectra at various temperatures.

In this paper, we show a schematic diagram for the deposition of PbS films in supplementary figure S1(a). Mobility data for PbS/graphene/glass are given in S1(b). The observed high mobility values of the films on graphene indicate that majority carriers (holes) of PbS films flow through graphene.

## 2 Experimental Design, Materials and Methods

PbS films were produced by chemical bath deposition (CBD) method using 180 mM lead nitrate (Pb(NO_3_)_2_, 99.3%; Kanto Chemical Co., Ltd., Japan), for Pb source, 90 mM thiourea (CS(NH_2_)_2_, 98%; Kanto Chemical Co., Ltd., Japan) for S source and 570 mM sodium hydroxide (NaOH, 98%; Daejung Reagent Chemicals & Metals Co., Ltd., South Korea) as complexing agent. Using a Teflon beaker containing 100 ml deionized water, NaOH bids were first put in, shortly after which Pb (NO_3_)_2_ was added to the solution and stirred using a magnetic stirrer for 10 min. CS(NH_2_)_2_ was added to the solution and finally the substrates were immersed vertically in the solution. The deposition was done at room temperature conditions and stirred at 180 RPM. The deposition time was 1–2 h [7].

A single layer of graphene was grown on a copper foil using CVD method. First, the Cu foil (99.8%, Alfa-Aesar, item no. 13382) was loaded into a quartz tube of the CVD system and annealed at 1000°C under 90 mTorr with flowing 10 sccm of H_2_ for 60 min in order to obtain the large-area crystalline surface of Cu without native oxide. The growth was initiated by a flowing reaction gas mixture (CH_4_/H_2_ = 15:10 sccm) under 560 mTorr for about half an hour, followed by rapidly cooling the film to room temperature (10°C min^−1^) under flowing H_2_ under 90 mTorr. The graphene/Cu film was coated with a thin layer of poly methyl methacrylate (PMMA). The Cu foil under the graphene was etched away in an aqueous solution containing 0.1M ammonium persulfate. After washing with deionized water several times, the PMMA/graphene was transferred onto a microscope slide glass substrate [8].

We successively deposited 1–5 layers of graphene on the microscope slide glass by the PMMA assisted layer-by-layer transfer process. To improve adhesion between graphene films and glass substrates, the substrates were thermally annealed to remove all residual PMMA. We learned that post-annealing was important for the adhesion of PbS films on graphene/glass. Graphene/glass were annealed at 600 °C for 2 h. An e-beam evaporator with a metal mask was used to deposit Ti electrodes on PbS/graphene/glass.

## Declaration of Competing Interest

The authors declare that they have no known competing financial interests or personal relationships which have, or could be perceived to have, influenced the work reported in this article.

## Appendix Supplementary materials

Image, application 1

Image, application 2

Image, application 3

Image, application 4

Image, application 5

Image, application 6

## References

[1] Di Bartolomeo A. (2016). Phys. Rep.

[2] Kim K.S., Zhao Y., Jang H., Lee S.Y., Kim J.M., Kim K.S., Ahn J.H., Kim P., Choi J.Y., Hong B.H. (2009). Nature.

[3] Liang F.X., Gao Y., Xie C., Tong X.W., Li Z.J., Luo L.B. (2018). J. Mater. Chem. C.

[4] Gautam M., Shi Z., Jayatissa A.H. (2017). Sol. Energy Mater Sol. Cells.

[5] Pan S., Liu X. (2012). J. Solid State Chem.

[6] Ampadu E.K., Kim J., Oh E., Lee D.Y., Kim K.S. (2020). Mater. Lett..

[7] Kim J., Ampadu E.K., Choi W.J., Oh E. (2019). Nanotechnology.

[8] Kim H.K., Kim Y., Kim K.S., Jeong H.Y., Jang A.R., Han S.H., Yoon D.H., Suh K.S., Shin H.S., Kim T.Y., Yang W.S. (2013). ACS Nano.

