# Supplementary material for: Data for direct chemical deposition of PbS on chemical vapor deposition grown-graphene for high performance photovoltaic infrared photo-detectors
Source: Data Brief. 2020 Sep 4;32:106273. doi: 10.1016/j.dib.2020.106273 (PMC7494446; doi:10.1016/j.dib.2020.106273)
Supplement: Supplementary file 6 [file mmc6.docx]

**Supplementary Figures**

**Data for direct chemical deposition of PbS on chemical vapor deposition grown-graphene for high performance photovoltaic infrared photo-detectors**

**Emmanuel K Ampadu ^a^, Jungdong Kim ^a^, and Eunsoon Oh^*a^**

**^a^***Department of Physics, Chungnam National University, Daejeon, Republic of Korea*

**Dong Yun Lee ^b^ and Keun Soo Kim^*b^**

**^b^***Department of Physics and Graphene Research Institute-Texas Photonics Center International Research Center (GRI-TPC IRC), Sejong University, Seoul, Republic of Korea*

[esoh@cnu.ac.kr](mailto:esoh@cnu.ac.kr) Fax: +82–(42)–822–5453 [kskim2676@sejong.ac.kr](mailto:kskim2676@sejong.ac.kr) Fax: +82–(23)–408–4316

1.
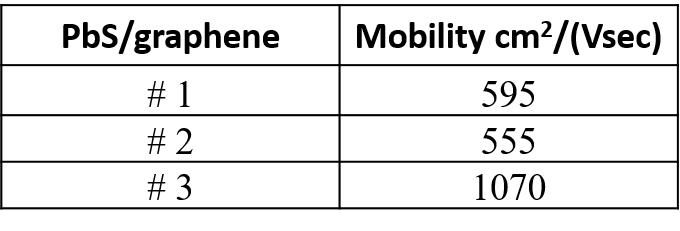

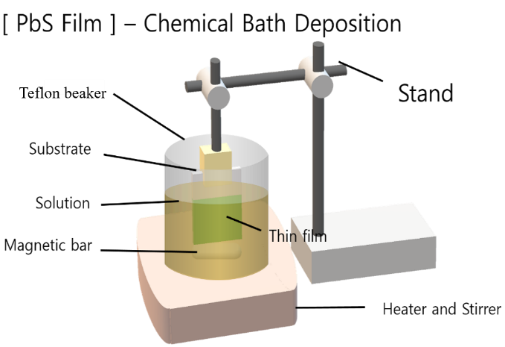
 (b)

Figure S1 (a) shows the schematic set-up for chemical bath deposition. Hall measurements of PbS films directly synthesized on graphene/glass were carried out using the van der Pauw method where Indium was used as a contact metal. Measured mobility values of three samples (#1 – #3) are presented in S1 (b). The recorded mobility was as high as 1070 cm^2^V^-1^s^-1^ at 300 K. We have reported a mobility value of 60 cm^2^V^-1^s^-1^ of p-type PbS films deposited on soda-lime glass at 300 K [1]. The high mobility value of PbS on graphene indicates that the majority carriers (holes) of PbS films flow through graphene.

References

[1] J. Kim, E. K. Ampadu, W. J. Choi, E. Oh, *Nanotechnology*, **30** (2019), 075201
